# Differential Performance and Lung Deposition of Levofloxacin with Different Nebulisers Used in Cystic Fibrosis

**DOI:** 10.3390/ijms23179597

**Published:** 2022-08-24

**Authors:** Carsten Schwarz, Claudio Procaccianti, Laura Costa, Riccardo Brini, Richard Friend, Grazia Caivano, Hosein Sadafi, Charles Mussche, Nicolas Schwenck, Michael Hahn, Xabier Murgia, Federico Bianco

**Affiliations:** 1 Division Cystic Fibrosis, CF Center Westbrandenburg, Campus Potsdam, Clinic Westbrandenburg, 14467 Potsdam, Germany; 2 Global Medical Affairs, Chiesi Farmaceutici S.p.A., 43122 Parma, Italy; 3 Global Technical Development, Chiesi Ltd., Chippenham SN14 0AB, UK; 4 Global Technical Development, Chiesi Farmaceutici S.p.A., 43122 Parma, Italy; 5 FLUIDDA nv, 2550 Kontich, Belgium; 6 PARI Pharma GmbH, 82319 Starnberg, Germany; 7 Independent Researcher, 48640 Berango, Spain

**Keywords:** levofloxacin, Quinsair, nebuliser, Zirela, eFlow, I-Neb, functional respiratory imaging, lung deposition

## Abstract

We compared the performance and levofloxacin (Quinsair) lung deposition of three nebulisers commonly used in CF (I-Neb Advance, eFlow rapid, and LC Plus) with the approved nebuliser Zirela. The delivered dose, delivery rate, and aerosol particle size distribution (APSD) for each device were determined using the methods described in the Pharmacopeia. High-resolution computed tomography scans obtained from seven adult patients with mild CF were used to generate computer-aided, three-dimensional models of their airway tree to assess lung deposition using functional respiratory imaging (FRI). The eFlow rapid and the LC Plus showed poor delivery efficiencies due to their high residual volumes. The I-Neb, which only delivers aerosols during the inspiratory phase, achieved the highest aerosol delivery efficiency. However, the I-Neb showed the largest particle size and lowest delivery rate (2.9 mg/min), which were respectively associated with a high extrathoracic deposition and extremely long nebulisation times (>20 min). Zirela showed the best performance considering delivery efficiency (159.6 mg out of a nominal dose of 240 mg), delivery rate (43.5 mg/min), and lung deposition (20% of the nominal dose), requiring less than 5 min to deliver a full dose of levofloxacin. The present study supports the use of drug-specific nebulisers and discourages the *off-label* use of general-purpose devices with the present levofloxacin formulation since subtherapeutic lung doses and long nebulisation times may compromise treatment efficacy and adherence.

## 1. Introduction

Cystic fibrosis (CF) is a multiorgan disease caused by the mutation of the cystic fibrosis conductance regulator (CFTR) gene, which encodes a membrane protein that regulates chloride and fluid transport across multiple epithelia and exocrine organs [1]. In the lungs, CF primarily manifests as an accumulation of thick airway secretions and a reduction in the mucus clearance rate, creating a favourable environment for the proliferation of opportunistic bacteria [2]. Once that bacterial colonisation with *Pseudomonas aeruginosa* is established, complete eradication is difficult to achieve [3], and the disease progresses to a state of chronic infection with concomitant inflammation and lung remodelling that produces structural changes and progressive, life-threatening deterioration of lung function [4]. *Pseudomonas aeruginosa* infections are predominant in adulthood and are a major cause of morbidity and mortality in people with CF [5].

Inhaled medications play a central role in the symptomatic management of CF pulmonary disease, enabling high local concentration while reducing systemic drug exposure [6]. CF patients are prescribed multiple inhaled therapies that usually include anti-inflammatory drugs (e.g., inhaled steroids), bronchodilators, mucoactive agents (e.g., nebulised hypertonic saline), and inhaled antibiotics [7]. Most of the drugs indicated for the treatment of CF are formulated as liquid solutions or suspensions and are therefore administered using nebulisers. Currently, two antibiotics (tobramycin and colistin) and the mucoactive mannitol are available as dry powders for inhalation [8]. 

Jet and vibrating membrane nebulisers are the most commonly used type of devices. Jet nebulisers use compressed air to break up liquids into respirable particles. In contrast, the aerosol generation principle with vibrating membrane nebulisers relies on the ultrarapid vibration of a membrane riddled with thousands of micron-size pores on top of the liquid reservoir that extrudes respirable aerosol droplets [9]. Traditional jet nebulisers have poor delivery efficiency, achieve relatively low lung deposition rates and are inconvenient to handle (not portable and noisy) [8]; however, they are still in use. Vibrating membrane nebulisers are the most widely used devices; they are noiseless, portable, usually have low residual volumes, and require shorter treatment times [10]. Moreover, smart vibrating membrane nebulisers equipped with adaptative aerosol technology only release aerosols when inhalation is detected (e.g., I-Neb), achieving high lung deposition rates (60–85%) [8]. The available nebulisers may vary from country to country, and the choice of the device may be decided by a person other than the patient or prescriber (e.g., healthcare policies) [11]. In addition, nebulised therapies impose a high treatment burden on people with CF, and patients are continuously making their own decisions in the self-management of the disease [12]. These factors may lead to *off-label* uses of nebulisers due to device preference or availability. In recent years, optimised drug-device combinations with vibrating membrane nebulisers have been approved for several inhaled antibiotics such as aztreonam, amikacin, and levofloxacin [5,13,14]. The *off-label* use of general-purpose nebulisers with these novel antibiotic formulations may have unpredictable results, potentially delivering either subtherapeutic doses with low-efficiency devices or toxic drug concentrations with highly efficient smart nebulisers. 

Levofloxacin is the first fluoroquinolone approved for inhalation in CF, providing an alternative mechanism of action to combat *P. aeruginosa* infections that complements the previously approved antibiotics for this indication: tobramycin, colistin, and aztreonam [6]. The dosage of inhaled levofloxacin is 240 mg (2.4 mL at 100 mg/mL) twice daily delivered with its drug-specific nebuliser Zirela in alternating cycles of 28 days on/off therapy [5]. We have recently shown that Zirela delivers respirable levofloxacin aerosols with a mass median aerodynamic diameter (MMAD) of 3.56 μm, requiring less than 5 min to deliver a full levofloxacin dose [15]. In the same work, a functional respiratory imaging (FRI) study that included the reconstructed lung models of 20 CF patients estimated a 37% intrathoracic deposition of levofloxacin with Zirela [15]. Since lung deposition largely depends on the device performance and aerosol characteristics, the *off-label* use of levofloxacin with nebulisers other than Zirela may be associated with inadequate levofloxacin dosing. Therefore, the present study was designed to investigate the nebuliser performance and levofloxacin lung deposition with a selection of general-purpose nebulisers commonly used in the management of CF: I-Neb Advance, eFlow rapid, and LC Plus. We first compared the delivered levofloxacin dose and aerosol particle size distribution (APSD) of the I-Neb Advance, eFlow rapid, and LC Plus nebulisers with the drug-specific Zirela device using the in vitro tests described in the Pharmacopeia. The delivered dose and APSD outcomes were then used as input parameters in FRI simulations designed to estimate the lung deposition of levofloxacin achieved by each device in the lungs of seven patients with mild CF.

## 2. Results

### 2.1. Delivered Dose and Delivery Rate of Nebulised Levofloxacin

**Figure 1:**
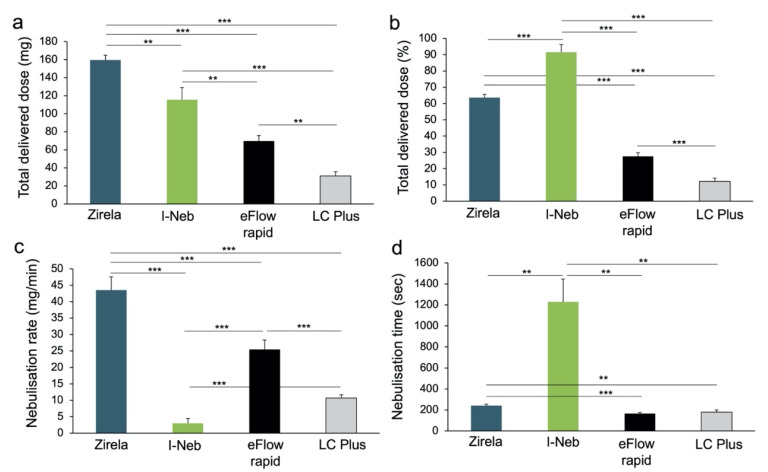
Outcomes of the delivered dose and delivery rate experiments. (**a**) Delivered dose, (**b**) percentage of the delivered dose, (**c**) nebulisation rate, and (**d**) nebulisation time for each nebuliser device. Experiments with Zirela, eFlow rapid and LC Plus were performed loading a full dose of levofloxacin (2.4 mL) into the nebuliser chamber. Experiments with the I-Neb were performed filling the nebuliser chamber with levofloxacin up to its maximum capacity (1.5–1.7 mL filling volume). Mean ± SD are shown. ** *p* < 0.01, *** *p* < 0.0001.

The delivered dose and delivery rate experiments revealed significant differences across nebulisers. Zirela achieved the highest mean delivered dose (159.6 ± 5.3 mg), and was significantly higher compared with the I-Neb (115.6 ± 13.3 mg, *p* < 0.01), eFlow rapid (69.6 ± 6.1 mg, *p* < 0.0001), and LC Plus nebulisers (30.9 ± 4.8 mg, *p* < 0.0001, Figure 1a). The lowest residual amount of levofloxacin was found after nebulisation with the I-Neb and Zirela nebulisers (7.4 ± 5.9 mg and 50.4 ± 5.0 mg, respectively). Conversely, more than half of the levofloxacin remained in the device after nebulisation with the LC plus (210.9 ± 6.2 mg) and eFlow rapid (154.5 ± 5.7 mg).

A low amount of levofloxacin was detected in the exhalation filter with the I-Neb (3.3 ± 1.3 mg) compared with the other nebulisers (LC Plus 11.5 ± 2.3 mg; eFlow rapid 28.6 ± 2.7 mg; and Zirela 50.4 ± 5.0 mg, all *p* < 0.0001). Therefore, the I-Neb exhibited the highest levofloxacin delivery efficiency (91.5% ± 4.7, Figure 1b), followed by Zirela (63.2% ± 1.9), eFlow rapid (27.5% ± 2.2), and LC Plus nebulisers (12.1% ± 1.8, all *p* < 0.0001). Nevertheless, the I-Neb showed the lowest nebulisation rate (2.9 ± 1.4 mg/min, *p* < 0.0001, Figure 1c), requiring more than 20 min on average to deliver a levofloxacin amount, which was still lower than the approved nominal dose (Figure 1d). On the contrary, Zirela registered the highest nebulisation rate (43.5 ± 4.1 mg/min) and was superior to the other nebulisers (*p* < 0.0001). The nebulisation time for the full content of a levofloxacin ampoule (240 mg) with Zirela was 242 s (~4 min). Nebulisation times were the lowest with the eFlow rapid and LC Plus nebulisers due to the high residual volumes of these devices.

### 2.2. Aerodynamic Particle Size Characterisation of Levofloxacin Aerosols

**Figure 2:**
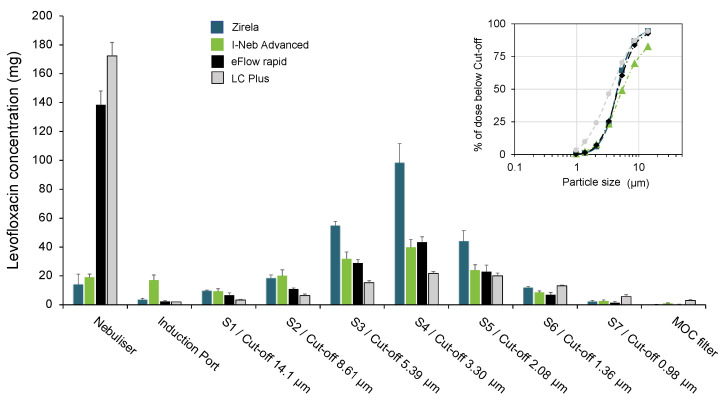
Next-Generation Impactor (NGI) stage (S) deposition of levofloxacin with each nebuliser. The inset shows the cumulative levofloxacin percentage below the size cut-off of each NGI stage. MOC, micro-orifice filter.

The levofloxacin aerosol deposition within the Next Generation Impactor (NGI) stages with each nebuliser is shown in Figure 2, and the parameters defining the APSD are displayed in Table 1. The LC Plus and eFlow rapid nebulisers showed the lowest emitted doses, with 65% and 43% of the nominal dose, respectively, remaining in the devices after nebulisation. Conversely, with Zirela and I-Neb nebulisers, less than 12% of the levofloxacin was detected in the device.

The highest MMAD was found for the I-Neb (5.0 ± 0.3 μm) and the lowest for the LC Plus (3.5 ± 0.2 μm). The Zirela and eFlow rapid showed similar MMAD values (4.5 ± 0.1 μm and 4.7 ± 0.2 μm, respectively) and lower geometric standard deviation (GSD) values than the other nebulisers, indicative of a more homogeneous particle size distribution of the aerosol plume. The LC Plus registered the highest fine particle fraction (FPF; 66.8%), followed by Zirela (58.1%), eFlow rapid (54.9%), and the I-Neb (44.8%). Nevertheless, Zirela registered markedly higher levofloxacin amounts in stages 3 (S3) to 5 (S5), which correspond with a cut-off size range from 5.39 μm (S3) to 2.08 μm (S5).

### 2.3. Functional Respiratory Imaging Assessment of Levofloxacin Lung Deposition

The lung deposition for each device with a nominal levofloxacin dose of 240 mg was simulated using FRI. In the case of the I-Neb, the theoretical delivered dose that would be achieved after nebulisation of a full levofloxacin ampule (2.4 mL) was estimated based on the mean delivered dose determined in vitro at its maximum filling volume. Thus, a delivered dose of 163.2 mg of levofloxacin was used for the I-Neb as an input parameter for the simulation.

**Figure 3:**
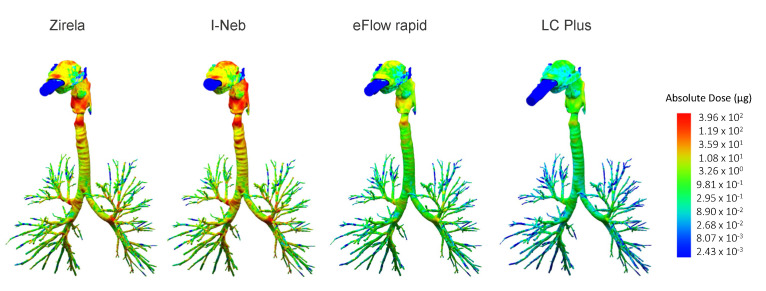
Differential levofloxacin deposition pattern across nebulisers in the three-dimensional reconstruction of the airway tree of a representative patient. The colour scale defines the absolute deposited levofloxacin in μg. The dashed line delineates the limit between extrathoracic and intrathoracic deposition.

**Figure 4:**
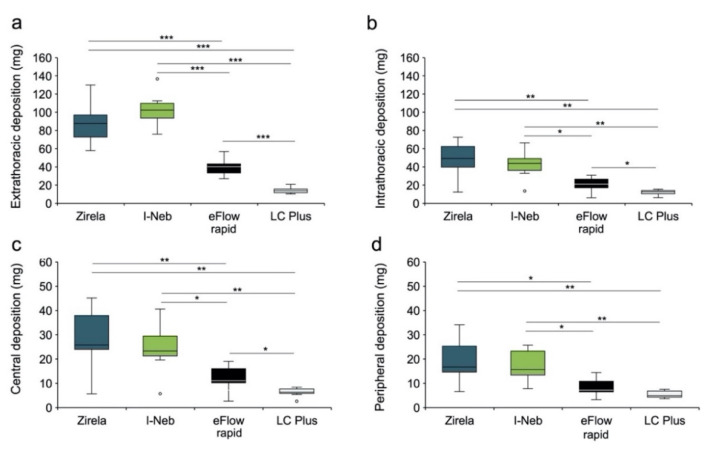
Levofloxacin lung deposition from functional respiratory imaging simulations. Mean (**a**) extrathoracic, (**b**) intrathoracic, (**c**) central, and (**d**) peripheral deposition for each nebuliser. Box plots show the lower quartile, median, and upper quartile, and the whiskers indicate the maximum and minimum values excluding outliers (outliers: values 3/2 times higher or lower than the upper or lower quartiles). * *p* < 0.05, ** *p* < 0.01, *** *p* < 0.0001.

The highest mean extrathoracic levofloxacin deposition values were found for the I-Neb (103.0 ± 19.4 mg) and Zirela (87.7 ± 24.6 mg; Figure 3 and Figure 4a). Zirela and I-Neb were superior in terms of intrathoracic deposition compared with the eFlow rapid and LC Plus nebulisers. The mean intrathoracic deposition with Zirela (48.2 ± 20.9 mg) was significantly higher than with either the eFlow rapid (20.6 ± 8.6 mg, *p* < 0.01) or LC plus nebulisers (11.7 ± 3.1 mg; *p* < 0.01, Figure 3 and Figure 4b). Similarly, the mean intrathoracic deposition with the I-Neb was 41.8 ± 16.5 mg, which was also significantly higher than with the eFlow rapid (*p* < 0.05) and LC Plus (*p* < 0.01). Accordingly, Zirela and I-Neb achieved a significantly higher central and peripheral levofloxacin deposition than the eFlow rapid and LC Plus nebulisers (Figure 4c,d).

It is worth noting that patient 4 had a narrower pharyngeal airway than the other patients (Supplementary Figure S1). In this patient, the intrathoracic levofloxacin deposition was low with all devices (Zirela 12.2 mg, I-Neb 13.5 mg, eFlow rapid 5.9 mg, and LC Plus 6.20 mg).

## 3. Discussion

Inhaled levofloxacin is the fourth antipseudomonal antibiotic approved for the treatment of people with CF. Clinical trials have demonstrated a significant improvement in lung function and a reduction in the exacerbation rate with inhaled levofloxacin against placebo [16,17] and equivalent outcomes compared with tobramycin in adult patients with CF [18]. Levofloxacin belongs to the fluoroquinolone antibiotic class and displays broad-spectrum activity against Gram-negative and Gram-positive bacteria, disrupting the bacterial DNA synthesis through inhibitory interactions with the DNA gyrase and topoisomerase IV enzymes [5,19]. The efficacy spectrum and mechanism of action of levofloxacin are different from the previously approved inhaled antipseudomonal antibiotics (tobramycin, colistin, and aztreonam). Therefore, levofloxacin complements the existing therapeutic repertoire against *P. aeruginosa* lung infections. In this regard, a recent observational, single-centre study conducted in a real-world setting has shown a significant improvement in lung function in CF patients that switched from either inhaled colistin or tobramycin to levofloxacin [20].

Inhaled levofloxacin has been approved to be delivered with the specific nebuliser Zirela and should not be delivered with other devices. However, several devices are available as general-purpose nebulisers, which may give rise to the *off-label* use of nebulisers [8]. Therefore, the present study was designed to compare the performance and levofloxacin lung deposition of four nebulisers, featuring different aerosol-generating principles (jet vs. vibrating membrane) and technologies (continuous vs. breath-coordinated nebulisation) commonly used in the symptomatic treatment of the CF pulmonary disease.

The compendial assessment of these devices revealed remarkable differences in performance. Although the eFlow rapid was superior to the LC Plus, both devices showed a low delivered dose due to their relatively high residual volumes, which respectively accounted for 87% and 64% of the nominal levofloxacin dose. The residual volume of the eFlow rapid is deliberately designed to mimic the LC Plus performance. These devices display high-volume drug chambers (e.g., LC Plus has a maximum fill volume of 8 mL) that enable the uninterrupted nebulisation of large drug volumes; however, they showed poor efficiency with levofloxacin, whose 240 mg dose is contained in a low volume (2.4 mL). On the contrary, the low residual volumes with Zirela and, particularly, with the I-Neb, enabled a higher levofloxacin delivery efficiency. Unfortunately, a head-to-head comparison of the total delivered dose between Zirela and I-Neb nebulisers was not feasible because in vitro experiments with the I-Neb were conducted with lower levofloxacin doses due to the low volume of the drug chamber of the I-Neb.

The main difference between Zirela and I-Neb is that Zirela delivers aerosols continuously, although it stores the aerosol generated during the patients’ exhalation in an aerosol chamber; the I-Neb operates with adaptative aerosol technology and releases aerosols only during 50 to 80% of the patient’s inhalation [21]. Consequently, the I-Neb showed the highest delivery efficiency across nebulisers. Notably, Zirela showed a delivered dose of 63%, while the theoretical maximum delivered dose for a 1:1 breathing pattern without a valved aerosol chamber would be 50%. Nonetheless, the high delivery efficiency with the I-Neb was achieved at the expense of the nebulisation rate; while Zirela delivered 160 mg of levofloxacin in approximately 4 min, the I-Neb required over 20 min to deliver just 115 mg. Therefore, using the I-Neb to administer levofloxacin twice a day, even at lower nominal doses, would significantly increase the treatment burden of patients with already intensive regimes. A survey reported that adult CF patients spent 108 min on average in treatment activities, with nebulisation activities requiring the longest time (41 min), followed by airway clearance (29 min), exercise (29 min), and oral treatments (9 min) [12]. Another study compared the lung function of 70 patients with stable CF who switched from a conventional jet nebuliser to the eFlow rapid vibrating membrane nebuliser. Although there were no significant differences in lung function parameters after one year on the new device, a mean reduction in the treatment time from 31.3 min/day with the jet nebuliser to 10.2 min/day with the eFlow rapid was reported and was associated with higher patient satisfaction [22]. Therefore, shortening inhaled treatments is of capital relevance in CF since it improves the patient quality of life and adherence to treatment [23].

The levofloxacin lung deposition was investigated using FRI. This method uses metadata from high-resolution CT scans obtained from actual CF patients to generate computer-aided, three-dimensional models of the airway tree, which are then used to run computational fluid dynamics (CFD) simulations to assess the patient-specific aerosol deposition [24]. FRI was first validated in a crossover study conducted in asthmatic patients who inhaled a radiolabelled tracer delivered with a vibrating membrane nebuliser and then underwent single-photon emission computed tomography (SPECT) to quantify the peripheral lung deposition [24]. The authors reported an excellent correlation between FRI and SPECT/CT, with less than 3% variation in lung deposition. Subsequent studies have shown good agreement between scintigraphy data and FRI simulations for dry powder inhalers (DPIs) and pressurised metered-dose inhalers (pMDIs) [25,26,27,28]. 

The aerosol particle diameter, airway size and condition of the lungs, and breathing flows are the three main factors determining the deposition of drug droplets in the respiratory tract [11]. Our FRI experiments covered these factors by including in the CFD simulation the APSD data obtained in vitro, actual CF breathing patterns, and the airway trees of people with CF. The use of several patient-specific airway tree models provided a solid framework to assess the lung levofloxacin deposition and inter-patient variability. The CF breathing pattern was adopted from Schwarz et al. and provides a more representative breathing pattern for the FRI simulation than the compendial one, which overestimates lung deposition [15] (Supplementary Figure S2). Although the breathing pattern averaged from CF patients has higher minute ventilation (23.5 L) than the compendial breathing pattern (7.5 L), the shorter inspiratory time yields a steeper slope of the inspiratory flow profile that better reflects the peak inspiratory flows during actual inhalation.

Zirela achieved the highest mean levofloxacin lung deposition (48.2 mg), closely followed by the I-Neb. Interestingly, the outstanding drug delivery efficiency of the I-Neb did not achieve the highest lung deposition, which can be explained by the outcomes of the APSD experiments. NGI tests revealed higher MMAD and GSD values and a relatively high levofloxacin deposition in the artificial throat (induction port) for the I-Neb than Zirela, which was reflected in FRI experiments by a higher extrathoracic and lower intrathoracic levofloxacin deposition with the I-Neb. Nevertheless, Zirela and I-Neb outperformed the other nebulisers in terms of lung dose, reaching 2- and 4-fold higher intrathoracic levofloxacin deposition than the eFlow rapid and LC Plus, respectively. The lung doses estimated for the eFlow rapid and LC Plus nebulisers can be regarded as low and certainly discourage the *off-label* use of these devices with the present levofloxacin formulation since local antibiotic doses below the minimum inhibitory concentration increase the risk of generating antibiotic-resistant bacterial strains [29], which have a negative impact on disease management and progression [5].

The intrathoracic deposition with Zirela represented 20% of the nominal dose. This value is lower than our previous FRI study performed with the same drug-device combination and applied to the same set of CF patients, in which the intrathoracic deposition reached 39.5% [15]. Although in vitro experiments showed similar outcomes for the delivered dose (164 ± 10 mg vs. 159.6 ± 5 mg in the present study) in both studies, major differences were found regarding the MMAD (3.54 μm vs. 4.5 μm). Such differences in MMAD may be explained by slight inter-laboratory variances in the NGI experimental protocol. Our previous study determined the APSD of levofloxacin with Zirela, operating the NGI at controlled relative humidity conditions of 50%. However, relative humidity was not controlled in the present study, which might have affected the shrinkage behaviour of aerosol particles, accounting for deviations in APSD measurements between studies. Since we used the APSD data from the present study as an input parameter for FRI simulations, the lung deposition values reported here represent a conservative approach to levofloxacin lung deposition that may underestimate the actual levofloxacin lung dose during inhalation at room temperature.

The present study has some limitations. First, in vitro studies with the I-Neb were conducted with lower levofloxacin amounts than with the other nebulisers because the maximum reservoir capacity of the I-Neb is lower than the volume of one levofloxacin ampoule. Subsequent refill with the solution remaining in the ampoule was avoided since it could introduce an additional variable to the analysis, which was not consistent with the assessment of the other devices. In addition, the algorithm of the I-Neb was changed from breath-coordinated aerosol release mode to a continuous aerosol delivery for the NGI measurements. We acknowledge that these variables could influence the delivered dose and APSD measurements. Secondly, the in silico character of FRI represents an intrinsic limitation of the method, and even though FRI considers the main factors affecting lung deposition, it has not been validated against lung imaging data in dedicated levofloxacin inhalation studies.

## 4. Materials and Methods

### 4.1. Nebulisers

Levofloxacin (Quinsair, Chiesi Farmaceutici, Parma, Italy) aerosols were generated with four nebulisers (Table 2). Zirela and eFlow rapid (both from PARI Pharma, Starnberg, Germany) are vibrating membrane nebulisers that continuously generate low-velocity aerosols and comprise a valved aerosol storage chamber that stores generated aerosols also during patients’ exhalation, thus minimising therapy time. The I-Neb Advance- (Philips Respironics, Chichester, UK) also uses the vibrating membrane principle to generate aerosols but operates with adaptative aerosol delivery (AAD) technology, and it only releases aerosols when inhalation is detected. Finally, the LC Plus nebuliser (PARI Pharma, Starnberg, Germany) is a jet nebuliser that uses compressed air to break liquids into aerosol particles.

### 4.2. Delivered Dose and Delivery Rate of Nebulised Levofloxacin

The delivered dose and the delivery rate of levofloxacin for each nebuliser were determined according to the guidelines described in the United States Pharmacopeia (USP) [30]. The set-up for each experiment consisted of the corresponding nebuliser, inspiratory and expiratory drug filters (PARI Filter valve set, Starnberg, Germany), and a breath simulator (BRS 3000, Copley Scientific, Nottingham, UK; Supplementary Figure S3) programmed with the sinusoidal adult breathing pattern described in the USP: tidal volume (V_T_) of 500 mL, rate of 15 cycles/min and inhlaltion:exhalation ratio (I:E) of 1:1. With the set-up in place, the content of one ampoule of levofloxacin (nominal dose 240 mg, ~2.4 mL) was loaded into the drug chamber of the corresponding nebuliser. The volume of one levofloxacin ampule exceeded the volume of the drug loading chamber of the I-Neb Advance-, and therefore, experiments with the I-Neb were performed, filling the chamber up to its maximum capacity (~1.5–1.7 mL). One minute after starting nebulisation, the inhalation drug collection filters were collected to determine the delivery rate. The filters were replaced, and nebulisation was resumed until the delivery of the full levofloxacin dose was completed. Experiments were performed with two independent nebulisers of each type in duplicate, accounting for a total of four repetitions with each nebuliser. All experiments were performed at 21 °C ± 2.

After nebulisation, the inhalation and exhalation filters with their filter housings were carefully rinsed with diluent (water:methanol 80:20 *v*/*v*) over a funnel placed over a 50 mL volumetric flask. The filters were thoroughly washed using the dilution solution and squeezed with tweezers to extract the maximum amount of levofloxacin. Similarly, the nebulisers were rinsed with diluent to determine the residual levofloxacin remaining in the devices. The amount of levofloxacin in the filters and the residual levofloxacin remaining in the nebulisers was determined by High-Pressure Liquid Chromatography (HPLC, Supplementary Method S1). The delivery rate (mg/min) refers to the amount of levofloxacin collected in the inhalation filter after 1 min of nebulisation, whereas the total delivered dose (in mg) refers to the sum amount of levofloxacin collected in the inhalation filter after 1 min plus the amount collected in the replaced filter after nebulisation of the full levofloxacin dose. The percentage of the delivered dose refers to the amount of levofloxacin collected in the inhalation filter divided by the total levofloxacin collected in all set-up components. The time required to nebulise the full levofloxacin dose was also registered (nebulisation time).

### 4.3. Delivered Dose and Delivery Rate of Nebulised Levofloxacin

The APSD was determined using the Next Generation Impactor (NGI, Copley Scientific, Nottingham, UK) [30]. The NGI was cooled at 5 °C for at least 90 min before starting each experiment (NGI Cooler, Copley Scientific, Nottingham, UK). The system was first checked for air leaks. A flow rate of 15 L/min was generated using the vacuum pump (TPK 2100, Copley Scientific, Nottingham, UK), which was confirmed using a calibrated volumetric flow meter (DFM 2000, Copley Scientific, Nottingham, UK). After set-up validation, the corresponding nebuliser was connected to the induction port with the appropriate mouthpiece adapter (Supplementary Figure S4). A full ampule of levofloxacin (240 mg at 100 mg/mL) was loaded into each nebuliser, and continuous nebulisation was started. For the I-Neb, the nebuliser chamber was filled up to its maximum capacity, and the nebulisation algorithm was changed from inhalation-triggered to continuous nebulisation.

After delivery of the full dose, the amount of levofloxacin deposited in the induction port, NGI stages (Stage 1 to Stage 7) and the micro-orifice collector (MOC) filter were extracted with diluent and determined by HPLC. The amount of levofloxacin remaining in the nebuliser was also determined. The MMAD, geometric standard deviation (GSD) and fine particle fraction (FPF) were calculated. All of the experiments were performed at 5 °C ± 3 with two independent nebulisers of each type in duplicate (*n* = 4 per device).

### 4.4. Functional Respiratory Imaging Assessment of Levofloxacin Lung Deposition

**Figure 5:**
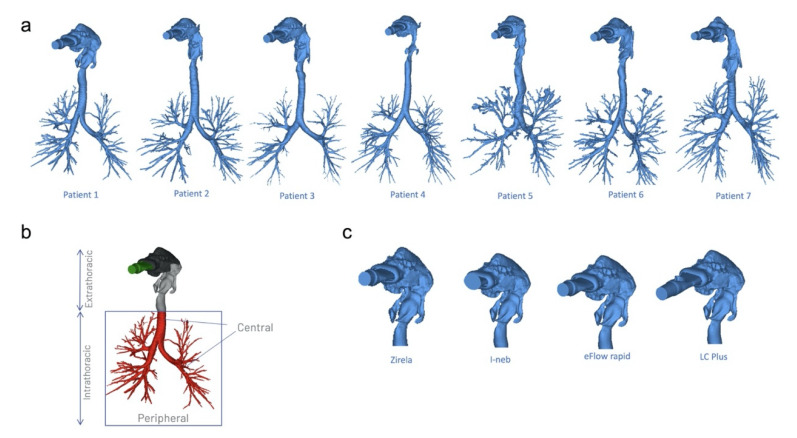
(**a**) Three-dimensional (3D) reconstructions of the airway tree from high resolution computed tomography (CT) scans obtained from seven patients with mild cystic fibrosis. (**b**) Deposition areas are divided into extrathoracic regions, comprising the mouth and upper airways, and the intrathoracic airways. The intrathoracic region is further divided into central airways, from the start of the trachea and including all the airways visible on the CT scans, and the peripheral lung regions. (**c**) The geometry of each device was reverse-engineered into a 3D computer-aided design model and then virtually coupled to the patients’ airway models.

The levofloxacin deposition in CF lungs was estimated by means of FRI. This technique uses metadata from high-resolution computed tomography (CT) scans from actual CF patients to render three-dimensional airway tree models, which are then used to run CFD simulations to estimate the lung deposition of well-characterised aerosols. The airway tree of seven adult patients with mild CF was digitally reconstructed into three-dimensional models (Figure 5a). Informed consent was obtained from each patient, and ethical approval was granted by the Institutional Review Board of the University Hospital in Antwerp, Belgium; file number: B300201731264. The patients’ characteristics have been recently described by Schwarz et al. [15]. Briefly, the male-to-female ratio was 5:2, the mean age was 23 years (range 18–37), and the mean percentage predicted forced expiratory volume at one second (ppFEV_1_) was 91% (range 72–109%). Hence, the disease stage was classified as mild CF according to the guidelines of the Cystic Fibrosis Foundation Patient Registry [31].

Segmentation and 3D model operations were performed in commercially available validated software packages (Mimics 20.0 and 3-Matic 12.0, Materialise nv, Ghent, Belgium). The airway tree (i.e., intraluminal air) could be segmented down to bronchi of about 1–2 mm in diameter. Beyond this point, the CT resolution is insufficient to distinguish alveolar and intraluminal air. Segmentation was semi-automatic, with airways then manually checked and missing branches added. A typical airway model includes 5–10 generations, depending mainly on the individual patient’s disease stage. The patient-specific models included the extrathoracic region, comprising the mouth and upper airways, and the intrathoracic airways, which were divided into (1) central airways, from the start of the trachea and including all the airways visible on a high-resolution CT scan, and (2) the peripheral airways (Figure 5b). The geometry of each device was reverse-engineered into a 3D computer-aided design model and then virtually coupled to the patient’s airway models (Figure 5c).

The MMAD, GSD, FPF, and the total delivered dose obtained from in vitro experiments were used as input parameters for the CFD simulations. In the case of the I-Neb, the theoretical delivered dose that would be achieved after nebulisation of a full levofloxacin ampule (2.4 mL) was estimated based on the delivered dose obtained with a filling volume of approximately 1.7 mL. A breathing pattern averaged from patients with mild CF consisting of a V_T_ of 759 mL, rate of 31 cycles/min, I:E 1:1.23, and a mean flow rate of 46.5 L/min, recently described by Schwarz et al. [15], was applied for the lung deposition simulations. The surface meshing strategy and the boundary conditions have been described elsewhere [32,33].

### 4.5. Statistical Analysis

The data are presented as mean ± standard deviation (SD). A *t*-test with Levene’s test for equality of variances was used for the head-to-head comparison between nebulisers (SPSS Statistics software version 23, IBM Corporation, Armonk, NY, USA). A *p* < 0.05 was accepted to determine a statistical significance.

## 5. Conclusions

The present study compared the performance and levofloxacin lung deposition of three nebulisers commonly used in the symptomatic treatment of CF (I-Neb, eFlow rapid, and LC Plus) with the drug-specific nebuliser Zirela. The compendial performance assessment showed remarkable differences across nebulisers. Zirela showed the best performance considering delivery efficiency, nebulisation time, and lung deposition, requiring approximately 4 min to deliver a full levofloxacin dose. The eFlow rapid and the LC Plus showed low delivery efficiencies, with most of the levofloxacin remaining in the device after aerosolisation due to their high residual volumes. The I-Neb Advance- showed the highest aerosol delivery efficiency but required exceptionally long nebulisation times, which were not associated with a higher lung dose than Zirela. The results from the present study support the use of the approved, drug-specific nebuliser and discourage the *off-label* use of general-purpose devices with the present levofloxacin formulation since subtherapeutic lung doses and long nebulisation times may, respectively, compromise treatment efficacy and adherence.

## Figures and Tables

**Table 1:** Emitted dose and aerosol particle size distribution parameters for each nebuliser.

Nebuliser	Recovered Levofloxacin (mg)	Emitted Dose (mg)	MMAD (μm)	GSD	FPF (%)
Zirela	258.1 ± 4.7	243.8 ± 5.8	4.5 ± 0.1	1.6 ± 0.1	58.1 ± 1.7
I-Neb Advance *	173.4 ± 13.9	154.3 ± 15.4	5.0 ± 0.3	1.9 ± 0.0	44.8 ± 2.3
eFlow rapid	262.2 ± 4.0	123.7 ± 8.6	4.7 ± 0.2	1.7 ± 0.1	54.9 ± 1.8
LC Plus	262.8 ± 6.4	90.5 ± 3.9	3.5 ± 0.2	2.1 ± 0.0	66.8 ± 2.5

* Experiments with the I-Neb were performed filling the drug chamber to its maximum capacity. In addition, the nebulisation algorithm was changed from inhalation-triggered to continuous nebulisation. MMAD, mass median aerodynamic diameter; GSD; geometric standard deviation; FPF, fine particle fraction.

**Table 2:** Device characteristics and reported uses in Cystic Fibrosis.

Device	Type of Device	Type of Nebulisation	Power Supply/Compressor	Reported Uses in CF *
Zirela	VM	Continuous	eBase controller	Levofloxacin
I-Neb Advance	VM	Breath-coordinated	Built-in battery	Colistin suspension Tobramycin solution (Off-label) Liposomal amphotericin B (Off-label) Dornase alpha (Off-label) Hypertonic saline (Off-label)
eFlow rapid	VM	Continuous	eBase controller	Colistin suspension Tobramycin solution (Off-label) Dornase alpha Hypertonic saline
LC Plus	Jet	Continuous	Pari Turboboy SX	Colistin suspension Tobramycin Hypertonic saline

* Reported uses adapted from [8]. For the I-Neb, the reported uses in CF refer to the previous I-Neb AAD system. VM, Vibrating Membrane; CF, Cystic Fibrosis.

## Data Availability

The datasets of the current study are available from the corresponding author on reasonable request. They are not immediately publicly available because they have been obtained by a private-funded research activity.

## Supplementary Materials

The following supporting information can be downloaded at: https://www.mdpi.com/article/10.3390/ijms23179597/s1.
