# Supplementary material for: Differential Performance and Lung Deposition of Levofloxacin with Different Nebulisers Used in Cystic Fibrosis
Source: Int J Mol Sci. 2022 Aug 24;23(17):9597. doi: 10.3390/ijms23179597 (PMC9455972; doi:10.3390/ijms23179597)

International Journal of *Molecular Sciences*

MDPI

Supplementary Materials

Differential Performance and Lung Deposition of Levofloxacin with Different Nebulisers Used in Cystic Fibrosis

Carsten Schwarz [1], Claudio Procaccianti [2], Laura Costa [2], Riccardo Brini [3], Richard Friend [3], Grazia Caivano [4], Hosein Sadafi [5], Charles Mussche [5], Nicolas Schwenck [6], Michael Hahn [6], Xabier Murgia [7] and Federico Bianco [2,*]

Supplementary Materials

Supplementary Figure S1. Structural differences between the airway trees reconstructed from patients 4 and 1 (patient 1 is representative for the other patients included in the study). Patient 4 had a narrow pharyngeal airway associated with a low intrathoracic levofloxacin deposition, irrespective of the nebuliser used.

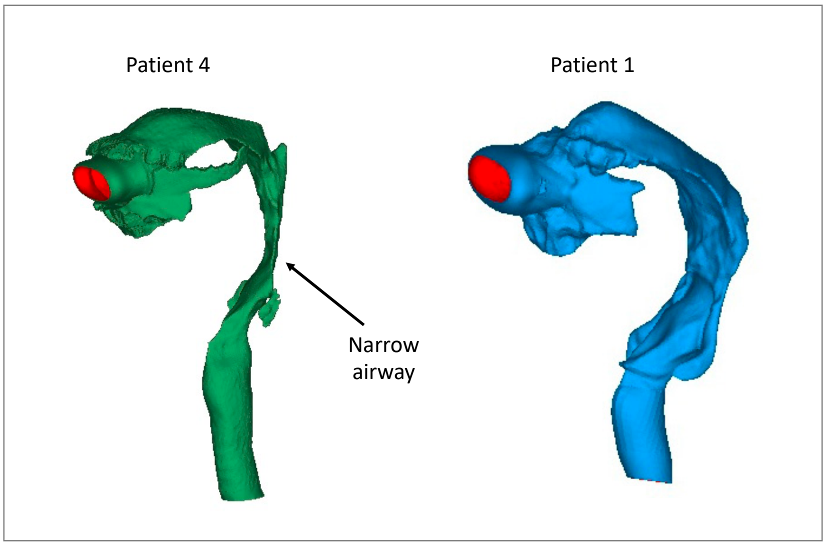

Supplementary Figure S2. Comparison of the intrathoracic levofloxacin deposition between the breathing pattern averaged from patients with mild CF (**Mild CF**: tidal volume [V_T] of 759 mL, rate of 31 cycles/min, inspiratory:expiratory ratio [I:E] 1:1.23) and the "adult" compendial breathing pattern that was used in the delivered dose and delivery rate experiments (**USP**: V_T of 500 mL, rate of 15 cycles/min, I:E 1:1). The intrathoracic deposition was estimated through functional respiratory imaging experiments conducted with the reconstructed airways of n=3 patients. Mean ± standard deviation. ** $p < 0.01$ (*t*-test).

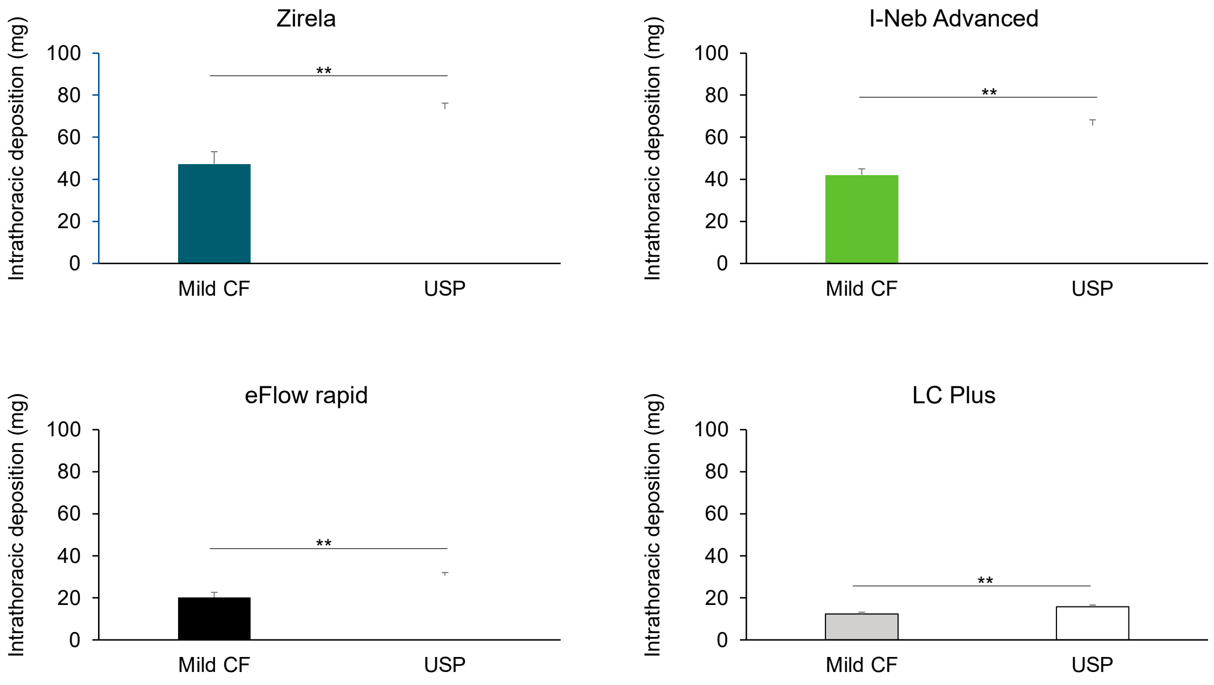

Supplementary Figure S3. Set-up to determine the delivered dose and delivery rate of levofloxacin according to the United States Pharmacopeia.

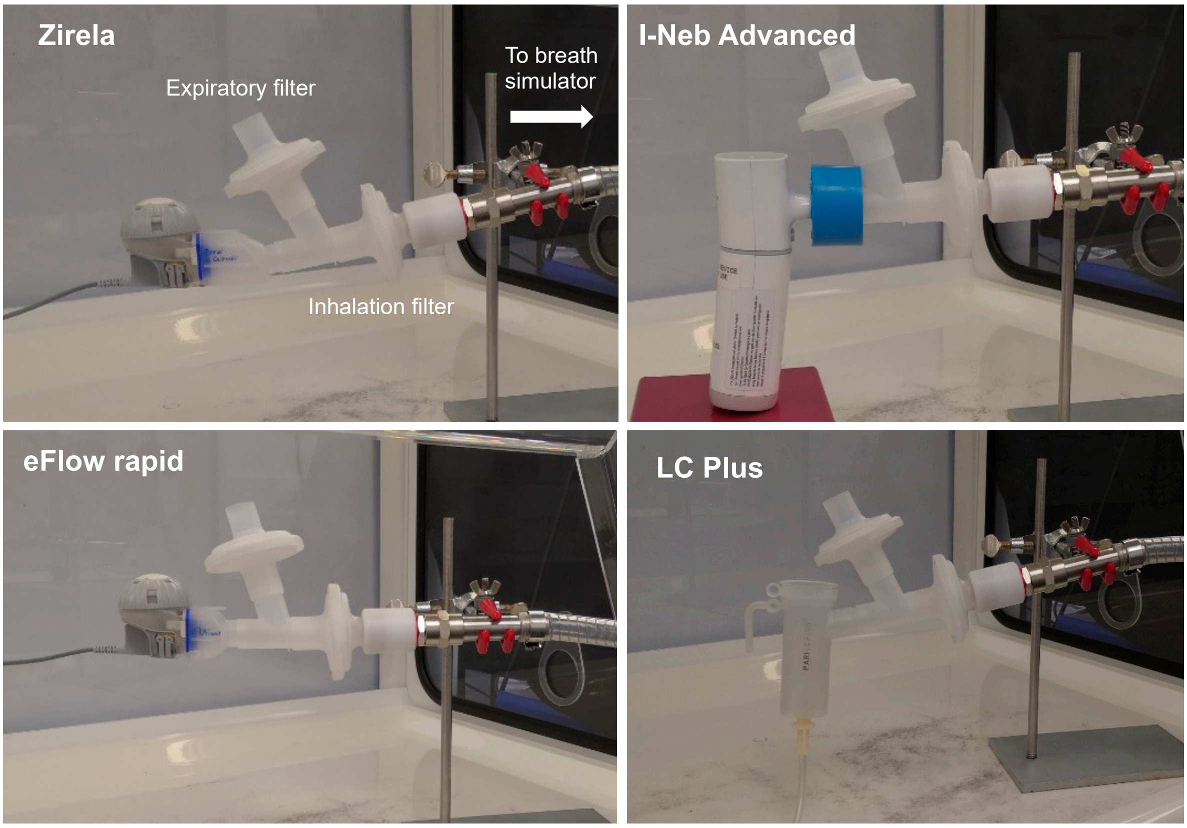

Supplementary Method S1. High-Pressure Liquid Chromatography (HPLC) method for levofloxacin detection. Levofloxacin was extracted from the collection filters and rinsed from the next generation impactor stages using an extraction diluent consisting of Water/Methanol (80:20, v/v). The chromatographic method is reported below.

Chromatographic conditions

HPLC device: Agilent HPLC 1260 Infinity II.
Detector: Diode Array or Variable Wavelength
Wavelength: 260 nm
Run time: 6 min
Column: Synergy Polar-RP 4µm, 80A, 150 x 3.0mm (Phenomenex)
Column temperature: 30ºC
Autosampler temperature:5ºC
Injection volume: 5 µl
Flow: 1 mL/min
Eluent A: 20mM potassium dihydrogen phosphate buffer pH 2.5
Eluent B: Methanol
Isocratic: Eluent A / Eluent B = 65/35 (v/v)
Rinse solution: Methanol/Water = 50/50 (v/v)
Retention time (RT): 4.1 min

Supplementary Figure S4. Set-up to determine the aerosol particle size distribution according to the United States Pharmacopeia.

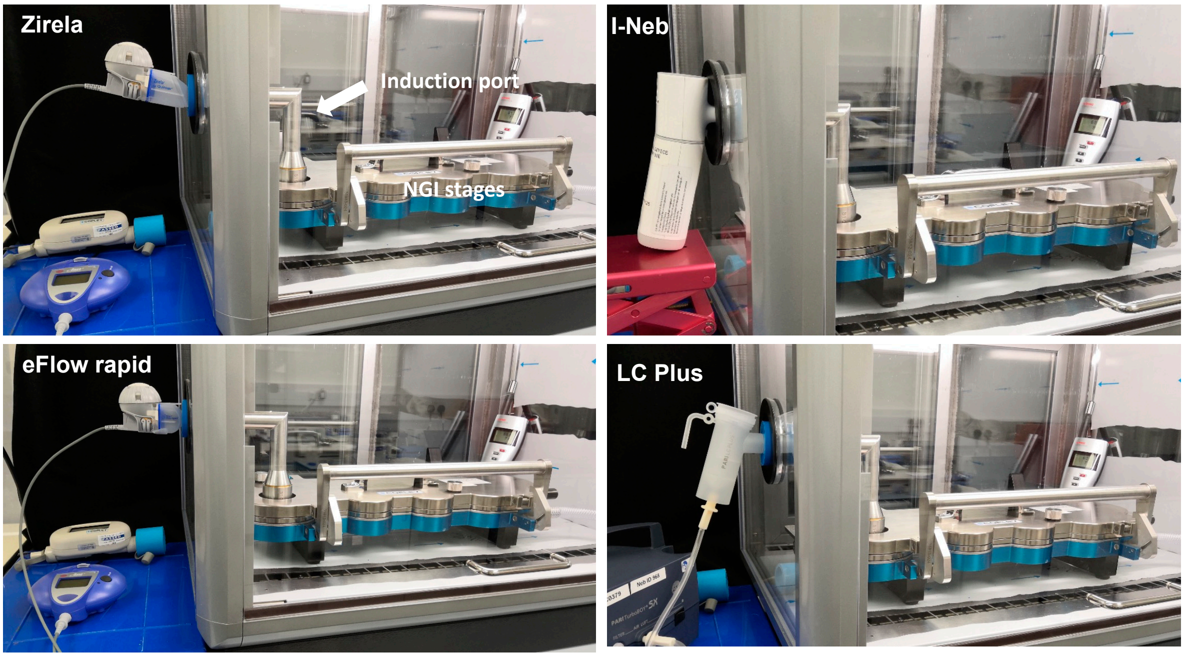

Supplement: Supplementary file 1 [file ijms-23-09597-s001.zip › ijms-1853714-supplementary.pdf]
